# Minimum Inhibitory Concentration of Glyphosate and of a Glyphosate-Containing Herbicide Formulation for *Escherichia coli* Isolates – Differences Between Pathogenicand Non-pathogenic Isolates and Between Host Species

**DOI:** 10.3389/fmicb.2019.00932

**Published:** 2019-05-03

**Authors:** Katrin Bote, Judith Pöppe, Roswitha Merle, Olga Makarova, Uwe Roesler

**Affiliations:** ^1^Institute for Animal Hygiene and Environmental Health, Freie Universität Berlin, Berlin, Germany; ^2^Institute for Veterinary Epidemiology and Biostatistics, Freie Universität Berlin, Berlin, Germany

**Keywords:** glyphosate, minimum inhibitory concentration, *Escherichia coli*, antimicrobial susceptibility testing, MIC distribution, statistical modeling

## Abstract

Glyphosate is the most extensively used herbicide in the world. However, concerns regarding its safety, side effects, and impact on other organisms have increased in recent years. This is the first study to analyze a large set of recent and historical *Escherichia coli* isolates varying in pathogenicity and beta-lactam resistance from different host species for their susceptibility to glyphosate isopropylamine salt (IPA), the active ingredient of the herbicide, and to a complete glyphosate-containing formulation (Roundup LB Plus). For this, minimum inhibitory concentrations (MIC) were determined for 238 *E. coli* isolates by broth microdilution in Mueller Hinton I media followed by the statistical analyses using Mann-Whitney-U test, multivariable analysis of variance (ANOVA) and a multivariable proportional-odds ordinal regression model. While the overall MIC distribution was narrow and lacked a highly resistant sub-population for both substances, statistical analyses revealed small but significant associations between glyphosate resistance levels and different factors tested. Mean MIC values for the entire dataset showed a higher level of resistance to the complete glyphosate-containing formulation (40 mg/ml IPA) than to pure glyphosate (10 mg/ml IPA) in *E. coli*. Isolates that originated from poultry had significantly higher MIC values for both pure glyphosate and the complete formulation. Pathogenic and non-extended-spectrum beta-lactamase (non-ESBL) *E. coli* isolates each showed significantly higher MIC values compared to commensals and ESBL-producing *E. coli* in pure glyphosate, but not in the complete formulation. Recently sampled isolates showed statistically higher MICs than the isolates of the historic standard *E. coli* collection of reference in pure glyphosate, when tested by nonparametric Mann-Whitney-U test, but not in the multivariable model. Further investigations are necessary to confirm whether these associations have a casual relationship with the glyphosate use or are the consequence of co-selection due to the increased application rates of antibiotics, heavy metals or other biocides. A possible accumulation of pathogenic bacteria in livestock animals fed with glyphosate-containing feed should also be considered.

## Introduction

The broad spectrum herbicide N-(Phosphonomethyl)glycine, commonly known as glyphosate, is one of the most-used pesticides in the world (Duke and Powles, 2008). It targets the enzyme 5-Enolpyruvylshikimate-3-phosphate Synthase (EPSPS) in the shikimate pathway and disrupts the formation of aromatic amino acids and other secondary plant compounds (Steinrücken and Amrhein, 1980, 1984). The pathway is present in plants, unicellular parasites, certain bacteria, and fungi but not in mammals (Herrmann and Weaver, 1999; Roberts et al., 2002). For a long time, this has been considered as a significant advantage regarding toxicity in comparison to almost all other pesticides (Benbrook, 2016). The presence of EPSPS in various microorganisms led to patenting the substance as a broad-range antimicrobial (William, 2002).

In 1996, glyphosate-resistant (GR) crops became commercially available on the market causing a paradigm shift in the herbicide use and agricultural management (Duke, 2017). From then on, glyphosate could be applied throughout the whole cultivation time without harming the desired plants and its use worldwide increased exponentially (15-fold) (Duke and Powles, 2009; Benbrook, 2016). Today, GR variants exist for example in soybean, corn, cotton, canola, alfalfa, and sugar beets (Cerdeira and Duke, 2006; Green, 2016), although none of those GR plants are cultivated in the European Union, where the release of genetically modified organisms into the environment is highly regulated (Federal Ministry of Food and Agriculture, 2013). However, the considerable utilization in other parts of the world leads to an accumulation of glyphosate residues.

In this context, glyphosate has been found in soy beans (Arregui et al., 2004; Lorenzatti et al., 2004; Bøhn et al., 2014), assumed to be one of the main sources for residues found in livestock feed (von Soosten et al., 2016).

The presence of contaminants in glyphosate-treated soy and maize exposes farm animals’ microbiota to the herbicide ingredients (Krüger et al., 2013a; Katholm, 2016). The possible effects of glyphosate on the intestinal bacteria has been discussed recently. Shehata et al. (2013) state that pathogenic bacteria from the poultry microbiome are more resistant to glyphosate than beneficial members *in vitro*. Kurenbach et al. (2015) also found an increased tolerance and changed antibiotic responses in their tested *Escherichia coli* and *Salmonella enterica* serovar Typhimurium strains after exposure to sub-lethal concentrations of a herbicide formulation.

*Escherichia coli* is not only an important zoonotic pathogen in livestock but also ubiquitous in the environment. It represents the majority of *Enterobacteriacae* and is an intensively studied model organism in research. Additionally, *E. coli* is one of the two gram-negative bacteria species used for biocide efficacy testing as an surrogate for similar enteric bacteria (European Chemicals Agency, 2018), and has even been used for screening of bacterial metabolites with herbicidal activities (Gasson, 1980).

Contaminated food is the main source for colonization and infection of humans and a risk factor for transferring antimicrobial resistance genes (Aarestrup et al., 2008). Therefore, the question arises if an exposure to glyphosate can lead to a shift in the microbiome favoring the shedding of especially pathogenic or antibiotic-resistant *E. coli*.

Until now, there has been no detailed survey to define the susceptibility of *E. coli* to glyphosate. Therefore, our study aimed to (i) screen different *E. coli* isolates of clinical, non-clinical and environmental origin for susceptibility to glyphosate and to a glyphosate-containing formulation; (ii) compare historical and recent isolates in regards to a development of resistance over the time as glyphosate use increased; (iii) to investigate whether there is a link between host species or antibiotic resistance and glyphosate susceptibility.

## Materials and Methods

### Biological Material

In total, 238 *E. coli* strains from different environments were analyzed.

We tested sixty-five *E. coli* isolates from the standard *E. coli* collection of reference (ECOR) (Ochman and Selander, 1984). This collection was established before the broad usage of glyphosate, thus representing the variations in *E. coli* at that time and is used as historic controls.

Ninety commensal *E. coli* isolates sampled in 2014 and 2015 were obtained from the German Federal Institute for Risk Assessment. They were characterized as non-pathogenic and evenly divided into poultry, pig, and cattle origin as well as into extended spectrum beta-lactamase (ESBL) and non-ESBL producing *E. coli* strains.

In addition, the German Federal Office of Consumer Protection and Food Safety provided 83 pathogenic *E. coli* isolates from clinical cases they collected in 2014 and 2015 for the GERMAP survey of antibiotic resistances of pathogenic bacteria isolates. Poultry, pig, and cattle isolates were equally represented. Forty-eight of the isolates were non-ESBL and 35 were ESBL *E. coli* (Table 1).

**Table 1:** Origin and distribution of the 238 tested *E. coli* isolates divided by different collections.

Origin	ECOR	Commensal *E. coli*			Pathogenic *E. coli*		
			
	Non-		Non-	in		Non-	in
	ESBL	ESBL	ESBL	total	ESBL	ESBL	total
Poultry	–	15	15	30	3	12	15
Pig	2	15	15	30	19	17	36
Cattle	3	15	15	30	15	17	32
Human	39	–	–	–	–	–	–
Primate	9	–	–	–	–	–	–
Dog	3	–	–	–	–	–	–
Sheep	2	–	–	–	–	–	–
Leopard	2	–	–	–	–	–	–
Bison	1	–	–	–	–	–	–
Giraffe	1	–	–	–	–	–	–
Goat	1	–	–	–	–	–	–
Cougar	1	–	–	–	–	–	–
Kangaroo Rat	1	–	–	–	–	–	–
in total	65	45	45	90	37	45	83


### Minimum Inhibitory Concentration (MIC) Testing

There are no standards for testing MICs of herbicides. Therefore, a susceptibility testing protocol according to Wiegand et al. (2008), which is in compliance with CLSI M07-A10 standards for antibiotic susceptibility testing, was established. Polystyrene 96-well plates with a conical bottom (Sarstedt GmbH, Nümbrecht, Germany) were used. Based on growth and killing dynamics of a representative *E. coli*, Mueller Hinton (MH) I medium was chosen (Oxoid GmbH, Wesel, Germany, CM0405). MICs for MH II can be found in the Supplements.

A 40% monoisopropylamine salt solution of glyphosate (GLY) (Sigma-Aldrich Chemie GmbH, Taufkirchen, Germany) and the glyphosate-containing commercial formulation Roundup LB Plus (RU) (German registration number 024142-00) were used. Concentration is indicated in mg/ml for the isopropylamine salt (IPA) of glyphosate. Serial dilutions ranged from 80 to 1.25 mg/ml for the pure substance and from 160 to 2.5 mg/ml for the commercial formulation. The prepared plates were stored at -80°C until usage.

For testing, overnight cultures were diluted to an OD_600_ of 0.5 (10^8^ cfu/ml), which were further diluted 1:100 before adding 5 μl into each well (equivalent to 5 × 10^4^ cfu, 5 × 10^5^ cfu/ml, respectively). Each isolate was tested in triplicates. The plates were aerobically incubated at 37°C for 16–20 h in a humidity chamber according to Walzl et al. (2012). The growth within the wells was determined visually with a mirror below the plate and a light above (SensiTouch by Sensititre).

### Statistical Analysis

For statistical analyses and calculations, IBM^®^ SPSS^®^ Statistics Version 24 was used. All MIC data were ranked in ascending order prior to analyses and checked visually for normal distribution. As MIC values of GLY showed sufficient normal distribution, the data of GLY could be fitted by an ANOVA approach. Regarding RU, the MIC values were not normally distributed and only included the three levels 20, 40, and 80. Thus, it was decided to regard these levels as ordinal categories and to fit a proportional-odds ordinal regression model. The influences in terms of isolation time (ECOR and recent isolates), collection (commensals and pathogens), ESBL-status and host (poultry, pig, cattle) on MIC values of GLY or RU, respectively, were tested using

(i) univariable nonparametric Mann-Whitney-U tests for not normally distributed data, and
(ii) a multivariable analysis of variance (ANOVA) for GLY, or
(iii) a multivariable proportional-odds ordinal regression model for RU

to determine different factors.

Two different statistical models for each substance were adapted containing different parameters. In the first model (Model A) the influence of the time of isolation, the ESBL-status and the host on either GLY or RU were investigated.

In the ECOR collection, there were only few livestock associated isolates (two *E. coli* from pigs and three from cattle). Most of the isolates originated from humans or exotic animals (Table 1). Therefore, we created a second model (Model B) without the ECOR collection, which investigated the influence of the collection (pathogen or commensal), the ESBL-status and the host (poultry, pig, cattle) on either GLY or RU.

All two-way-interactions between influence factors were included in the initial models and removed if not statistically significant.

*P*-values below 0.05 were regarded as statistically significant. Model diagnostics included check for normality and homoscedasticity of residuals. For analysis of variance, the assumption of equal variances was also investigated. For proportional odds ordinal regression models, the assumption of proportionality as well as the assumption of parallel lines were additionally checked.

To obtain an epidemiological cutoff, MIC_95_ was calculated for GLY and for RU each.

## Results

**FIGURE 1:**
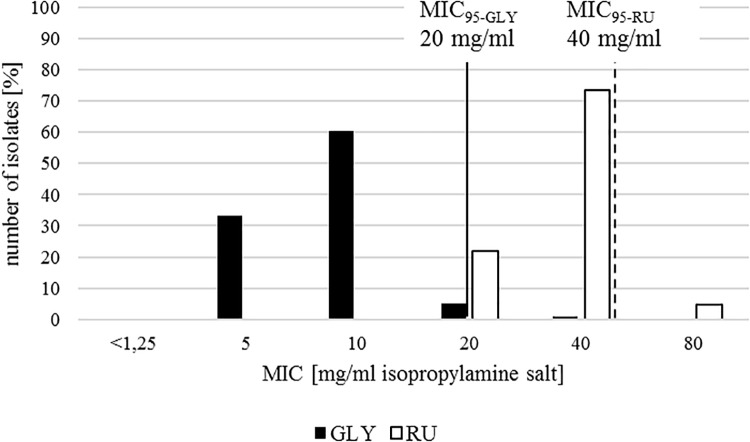
*In vitro* susceptibility profile of 238 *E. coli* isolates for glyphosate isopropylamine salt in a pure solution (GLY, black) and in Roundup LB Plus (RU, white). Minimum inhibitory concentration including 95% of all isolates (MIC_95_) is represented with a continuous line for GLY and a dashed line for RU.

Overall, MICs of glyphosate isopropylamine salt (GLY) and of the commercial herbicide formulation Roundup LB Plus (RU) were narrowly distributed with a clear segregation between both. In most of the isolates, growth was inhibited at a concentration of 10 mg/ml GLY (equating 7.41 mg/ml pure glyphosate) or 40 mg/ml RU (equating 29.63 mg/ml pure glyphosate), both representing the mean and the mode (Figure 1).

**FIGURE 2:**
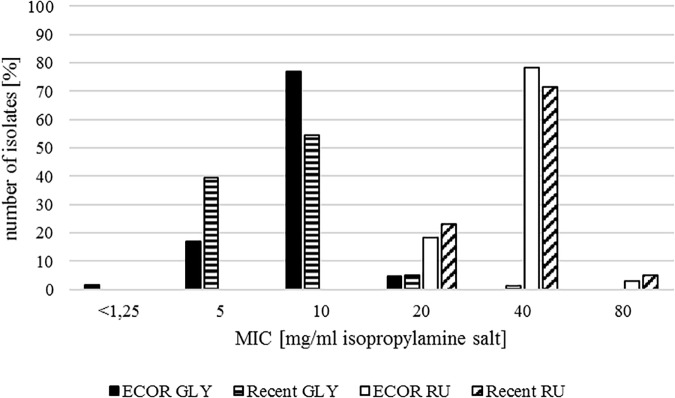
MIC for glyphosate isopropylamine salt for the ECOR collection (ECOR GLY, black) and the recently sampled isolates (Recent GLY, black with white stripes) and for the formulation Roundup LB Plus for the ECOR collection (ECOR RU, white) and the recently sampled isolates (Recent RU, white with black oblique stripes), respectively.

Most of the isolates from the ECOR collection showed a MIC of 10 mg/ml for GLY, which represented the mode and the median. For the herbicide formulation RU, the majority of the isolates had MIC values of 40 mg/ml. Overall MICs ranged from <1.25 to 20 mg/ml for GLY and 20 to 80 mg/ml for RU (Table 2 and Figure 2).

**Table 2:** MIC values of 238 *E. coli* for monoisopropylamine glyphosate salt (IPA) represented either as a pure solution (GLY) or as a part of the complete formulation Roundup LB Plus (RU).

MIC [mg/ml IPA]	ECOR		recent isolates		Commensal *E. coli*		Pathogenic *E. coli*		ESBL		non-ESBL		*E. coli* in total	
							
	GLY	RU	GLY	RU	GLY	RU	GLY	RU	GLY	RU	GLY	RU	GLY	RU
<1,25	1.5% (1)	0% (0)	0% (0)	0% (0)	0% (0)	0% (0)	0% (0)	0% (0)	0% (0)	0% (0)	0.7% (1)	0% (0)	0.4% (1)	0% (0)
5	16.9% (11)	0% (0)	39.3% (68)	0% (0)	48.9% (44)	0% (0)	28.9% (24)	0% (0)	45.8% (38)	0% (0)	26.5% (41)	0% (0)	33.2% (79)	0% (0)
10	76.9% (50)	0% (0)	54.3% (94)	0% (0)	47.8% (43)	0% (0)	61.5% (51)	0% (0)	51.8% (43)	0% (0)	65.2% (101)	0% (0)	60.5% (144)	0% (0)
20	4.6% (3)	18.5% (12)	5.2% (9)	23.1% (40)	3.3% (3)	22.2% (20)	7.2% (6)	24.1% (20)	2.4% (2)	24.1% (20)	6.5% (10)	20.7% (32)	5.0% (12)	21.9% (52)
40	0% (0)	78.5% (51)	1.2% (2)	71.7% (124)	0% (0)	74.4% (67)	2.4% (2)	68.7% (57)	0% (0)	73.5% (61)	1.3% (2)	73.6% (114)	0.8% (2)	73.5% (175)
80	0% (0)	3.1% (2)	0% (0)	5.2% (9)	0% (0)	3.3% (3)	0% (0)	7.2% (6)	0% (0)	2.4% (2)	0% (0)	5.8% (9)	0% (0)	4.6% (11)


*The tested isolates were divided into different groups. The ECOR collection served as an example of historic isolates as opposed to recent isolates (consisting of commensal and pathogenic isolates gathered in 2014 and 2015) or separated according to the susceptibility against beta-lactam antibiotics. Indicated as percentage share rounded to one decimal place after the point with the number of isolates in brackets.*

**FIGURE 3:**
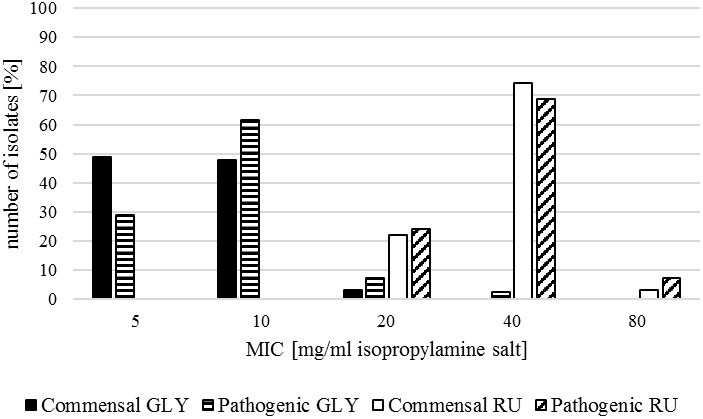
MIC for glyphosate isopropylamine salt for the commensal *E. coli* (Commensal GLY, black) and the pathogenic *E. coli* isolates (Pathogenic GLY, black with white stripes) and for the formulation Roundup LB Plus for the commensal *E. coli* (Commensal RU, white) and the pathogenic *E. coli* isolates (Pathogenic RU, white with black oblique stripes), respectively.

The commensal isolates of the investigated strains showed mostly a MIC of 5 mg/ml (representing the mode) or 10 mg/ml (representing the median) for GLY with a total range from 5 to 20 mg/ml. RU inhibited the growth of most strains at 40 mg/ml with a total range from 20 to 80 mg/ml (Table 2 and Figure 3).

In contrast to commensal isolates, pathogenic *E. coli* mostly showed a MIC of 10 mg/ml for GLY with a total range of 5–40 mg/ml. For RU, the MIC was in the range of 20–80 mg/ml, whereby 40 mg/ml was the most common minimal inhibitory concentration (Table 2 and Figure 3).

MIC_95_ representing 95% of the studied population was 20 mg/ml in GLY and 40 mg/ml in RU. For GLY there are two pathogenic *E. coli* isolated from cattle with a higher MIC than the cutoff. For RU 11 isolates (two from the ECOR collection isolated from humans, three commensal and five pathogenic *E. coli* from poultry and one pathogenic isolate from a pig) showed a MIC above the MIC_95_. All of the isolates belong to the non-ESBL group.

### Statistical Analysis

To test for differences between isolate parameters in glyphosate sensitivity, nonparametric Mann-Whitney-U test and depending on data distribution, two different statistical models were used.

In the Mann-Whitney-U test, both for GLY and RU, there were highly significant differences in MICs between the isolates from poultry (*P* < 0.01) compared to pig and cattle isolates which had lower MICs (Table 3).

**Table 3:** Effect of different parameters on MIC by means of univariable nonparametric Mann-Whitney-U test.

Comparison of		*P*-value	
		
		GLY	RU
Recent isolates	Historic isolates	**0**.**014**	0.667
ECOR collection	Commensal *E. coli*	**<0**.**001**	0.623
ECOR collection	Pathogenic *E. coli*	0.498	0.780
Pathogenic *E. coli*	Commensal *E. coli*	**0**.**004**	0.861
Non-ESBL	ESBL	**0**.**018**	0.362
Poultry	Pig	**<0**.**001**	**0**.**004**
Poultry	Cattle	**0**.**001**	**0**.**007**
Pig	Cattle	0.078	0.627


*Statistically significant *P*-values <0.05 are in bold. Parameters with higher MICs are underlined.*

Furthermore, more factors showed significant influence on GLY. Historic isolates from the ECOR collection had significantly lower MIC values (*P* < 0.05) than the isolates collected in the years 2014 and 2015. Pathogenic isolates differed highly significantly (*P* < 0.01) from the commensal isolates (with higher MIC values in the pathogenic group). Likewise, isolates classified as non-ESBL had statistically significantly higher MICs than the ESBL isolates (*P* < 0.05).

Model A included time of isolation (historic and recent), ESBL-status and host, whereas Model B (with the excluded ECOR strains) considered isolation as commensal or pathogen, ESBL-status and host (Table 4).

**Table 4:** *P*-values of the statistical models for glyphosate isopropylamine salt pure (GLY) (multivariable analysis of variance) and in Roundup LB plus (RU) (multivariable proportional-odds ordinal regression).

GLY		*P*-value		RU		*P*-value	
			
Comparison of		Model A	Model B	Comparison of		Model A	Model B
Recent isolates	Historic isolates	0.726	–	Recent isolates	Historic isolates	0.293	–
Pathogenic *E. coli*	Commensal *E. coli*	**–**	**<0**.**001**	Pathogenic *E. coli*	Commensal *E. coli*	**–**	0.314
Non-ESBL	ESBL	**0**.**013**	**0**.**035**	Non-ESBL	ESBL	0.443	0.479
Poultry	Pig	**<0**.**001**	**<0**.**001**	Poultry	Human	**0**.**031**	**–**
Poultry	Cattle	**0**.**01**	**0**.**002**	Poultry	Pig	**–**	**0**.**001**
Pig	Cattle	0.608	0.229	Poultry	Cattle	–	**0**.**002**


*Model A investigates the time point of isolation, the ESBL-status and the host, Model B (without the ECOR collection) investigates the following categories: pathogenic or commensal, the ESBL-status and the hosts (poultry, pig, and cattle). Statistically significant *P*-values <0.05 are shown in bold. Group with higher minimum inhibitory concentrations are underlined.*

In contrast to the results of the Mann-Whitney-U test for GLY, no difference between the strains of the ECOR collection and recent sampled isolates was seen in model A (*P* = 0.726).

However, the ESBL-status and the host species of the isolates showed statistically significant influence on the MIC values (*P* = 0.013 and *P* < 0.001). In agreement with the Mann-Whitney-U test, non-ESBL isolates had significantly higher MIC values compared to ESBL-positive isolates.

Tukey *post hoc* analysis for the hosts revealed significant differences between isolates from poultry and pigs (*P* < 0.01) and poultry and cattle (*P* = 0.01) with higher MICs in the poultry each, as well as between isolates from pigs and human (*P* = 0.019) and pigs and other species (*P* = 0.006) with lower MICs in pigs each. There was no significant difference between the isolates from pigs and cattle (*P* = 0.608).

Model B classified the differences between ESBL and non-ESBL isolates (*P* = 0.035) as well as between the hosts as significant. In accordance with model A, non-ESBL isolates and isolates from poultry had significantly higher MIC values than the ESBL isolates and isolates from cattle and pig. Additionally, a significant interaction between pathogenic and commensal isolates was present (*P* < 0.001). Pathogenic *E. coli* isolates showed significantly higher MIC values than commensals.

In the *post hoc* analysis, the differences between poultry and cattle (*P* = 0.002) as well as between poultry and pigs (*P* < 0.001) were clearly visible, with *E. coli* isolates from poultry showing significantly higher MIC values for glyphosate than isolates from other hosts.

For RU in model A, there was no significant difference between the strains of the ECOR collection and recently sampled isolates (*P* = 0.293), nor between ESBL and non-ESBL isolates (*P* = 0.443). However, a significant difference was found between poultry and human isolates (human isolates served as a reference category, *P* = 0.031). The Nagelkerke R^2^ in this model was 0.088, meaning that only a small proportion of the variance could be explained with this model.

Model B also showed no significant differences between ESBL and non-ESBL (*P* = 0.479) nor between commensal and pathogenic isolates (*P* = 0.314). Nevertheless, there was a significant difference between the hosts, i.e., between cattle and poultry (*P* = 0.002) and pigs and poultry (*P* = 0.001). Poultry served as reference category and had the highest MIC values compared to cattle and pigs. With a Nagelkerke’s R^2^ of 0.111, it still only explained a small proportion of the variance. Obviously, the variables included in the model were not the most important influence factors on the MIC values of the investigated *E. coli* strains.

## Discussion

After introducing GR plants two decades ago, glyphosate is now the most used herbicide in the world. Concurrently, concerns about possible resistances to glyphosate came to the fore. However, there is little information available about the sensitivity of naturally occurring *E. coli* to glyphosate.

This is the first broad study to systematically analyze 238 different *E. coli* isolates for their susceptibility not only against GLY alone but also against a glyphosate-containing herbicide formulation.

In our study, we found differences between GLY and RU with a 4-times higher median and mode in the latter. In contrast to prior findings in the literature, where herbicidal formulations were more toxic to bacteria (Clair et al., 2012; Mesnage et al., 2014), higher concentrations of RU were needed to inhibit bacterial growth.

However, it is difficult to compare the values to the few published data. Various glyphosate formulations are used, which makes it almost impossible to compare the obtained results as Mesnage et al. (2015) also point out. Pure glyphosate acid in particular has a low solubility (12 g/l) and is therefore not commercially used, but the isopropylamine glyphosate salt is present in most of the formulations (usually combined with a surfactant and water) (Giesy et al., 2000). After application and uptake, the salt dissociates and the free glyphosate acid translocates in the plant and inhibits EPSPS (Williams et al., 2000).

Additives and surfactants in formulations vary and manufacturers are not required to declare them publicly. This leads to complex mixtures with additional effects of the supplements themselves or interactions between all ingredients. Moreover, product compositions vary from brand to brand and regionally. Several formulations in experiments found in the literature contain the surfactant tallowamine. This substance is not used on the German market anymore and thus not present in our tested formulation (Senate Department for the Environment Transport and Climate Protection, 2012). The LD_50_ of tallowamine is much lower (oral 620 mg/kg rat) (Chemcas, 1997) than of pure glyphosate (oral 4873 mg/kg rat) (Chemcas, 2004) and indeed it has been shown that supplements in herbicide formulations can be more toxic than the active ingredient itself (Tsui and Chu, 2003; Mesnage et al., 2014; EFSA, 2015). This suggests that not only the activity of glyphosate, but rather the sum of different ingredients in a formulation or even some additives alone interact with the bacteria and influence the MIC. In response to this, a study by Clair et al. (2012) conducted with three food microorganisms observed differences between two different formulations and a glyphosate solution. In their tests, the formulations Roundup R400 and R450 were more toxic than the pure substance. Additionally, the effects were also disproportional to the amount of the active ingredient, proving the influence of additives and different mixtures.

Furthermore, in most studies only single or few isolates, which are often scarcely specified, were tested. For example, Kurenbach et al. (2015) published a MIC of 7.4 mg/ml for *E. coli* JB578 with the formulation Roundup Weedkiller in LB broth, similar to values with GLY from our experiment but not with RU. Shehata et al. (2013) published for *E. coli* a lower MIC of 1.2 mg/ml with a formulation called Roundup UltraMax. This formulation contained the surfactant tallowamine (Senate Department for the Environment Transport and Climate Protection, 2012), which, as mentioned above could be responsible for low MICs. Two *E. coli* isolates from Nielsen et al. (2018) had a comparable MIC of 20 mg/ml in reinforced clostridial medium or 80 mg/ml in brain heart infusion broth after anaerobic incubation in 96-well plates.

Besides the different MIC values, it is not always clear if stated concentrations in the literature are for glyphosate itself or the salt in a formulation and not all studies informed which media they used and how the susceptibility testing was conducted.

Specifically in nutrient rich media, bacteria may assimilate a certain amount of missing aromatic amino acids from their environment, bypassing the glyphosate-effects and thus tolerate higher concentrations. In medium lacking of aromatic amino acids, the MIC for glyphosate could be increased by adding them, which partly reversed the inhibition-effect of the herbicide (Haderlie et al., 1977; Nielsen et al., 2018).

In addition, glyphosate is known to be a chelator of bivalent cations (Madsen et al., 1978; Motekaitis and Martell, 2006). In cation-rich media, the active ingredient can be bound due to chelation leading to less free available active compounds. In MH I, the MIC was often one dilution step lower than in the cation-adjusted MH II (Supplementary Figure 1), with significant differences between MIC values in both media. However, differences between MIC for GLY and RU and difference between groups decreased (Supplementary Table 1).

Therefore, possible influences on MIC determination for glyphosate or glyphosate-containing formulations in general need further investigation, similarly concluded by Nielsen et al. (2018).

MIC_95_ has been used to distinguish different subpopulations by calculating epidemiological cutoffs values (ECV). No clear gap between isolates could be seen; nevertheless, there was a small subpopulation with less susceptibility. Besides overexpression of efflux pumps (Staub et al., 2012), changes in the EPSPS has been described as a reason for glyphosate resistance (Stalker et al., 1985; Eschenburg et al., 2002; Fei et al., 2013). Distribution of the isolates with a MIC above the cutoff reflect mostly the less susceptible categories in the statistical analysis (pathogenic, poultry origin, non-ESBL). Interestingly, the two isolates above the cutoff for GLY are not present in the group for RU, confirming again the varying behavior of formulations.

However, given the narrow distribution of all the MICs and an increase in absolute terms only one dilution step above the calculated cutoff, these isolates would need further investigation to determine a genetic basis of a glyphosate tolerance. Moreover, without a normal distribution, the calculated cutoff values might not reflect the real division between phenotypically resistant and sensitive populations (Lockhart et al., 2017).

### Historical and Recent Isolates

To determine if the sensitivity to glyphosate changed over time, we included the ECOR collection in our screening (Ochman and Selander, 1984). This gave us the possibility to compare isolates prior to and after the large-scale use of glyphosate that accompanied the introduction of genetically modified crops in the nineties (Cerdeira and Duke, 2006; Duke and Powles, 2009).

In the nonparametric test, we could see significant differences between the ECOR isolates and the isolates from recent years for GLY. This gap was due to differences between the ECOR and the commensal collection, rather than the ECOR and pathogenic isolates. However, the statistically significant difference could not be confirmed in the ANOVA model, which excluded the non-livestock associated isolates of the ECOR collection. Thus, it seems that the factor of isolation time (and therefore the span of glyphosate usage in general) is not one of the important influences on MIC against glyphosate in the dataset. Nevertheless, a tendency to higher MICs in the recent isolates appeared.

Sub-lethal concentrations of biocides and herbicides can lead to adaptation and increased resistance (Thomas et al., 2000; Capita et al., 2014) and may thus explain the differences. Increased MICs could further be a result of co-induction or co-selection of applied antibiotics, other biocides or heavy metals (Karatzas et al., 2007; Capita et al., 2014; Molina-González et al., 2014; Yazdankhah et al., 2014), especially as these antimicrobials were also intensively used in the last decades. There is no possibility to review, if recent isolates were exposed to glyphosate in the intestine of the host or in the environment. Data about residues in feed are missing and would for sure vary within the data set. In addition, only very few isolates from the ECOR collection are livestock-associated.

The statistical analyses with the formulation RU showed no significant differences between the ECOR collection and recently sampled isolates. It seems to be more difficult to become less susceptible against a complex formulation with various effects. Further, the MICs for RU are naturally higher than for GLY.

Overall, it is difficult to compare the strains of the ECOR collection and recent *E. coli* isolates in our livestock-related context, as ECOR lacks representative bacteria from livestock. Whether the observed difference in sensitivity to GLY is based on the increased use of glyphosate, antibiotics or other compounds in recent years, is yet to be established. In order to prove a change in susceptibility over time, historic isolates from farm animals should be investigated in future studies.

### Commensal and Pathogenic Isolates

In previous studies, it was discussed that pathogenic bacteria are likely to be more tolerant to glyphosate (Krüger et al., 2013b; Shehata et al., 2013). Shehata et al. (2013) found higher MIC values in strains of pathogenic species like different *Salmonella* serovars and *C. perfringens* compared with, e.g., enterococci or lactobacilli. Krüger et al. (2013b) confirmed the differences in sensitivity between Clostridia and enterococci. However, pathogenic and non-pathogenic strains of the same bacteria species were never investigated.

To assess this, we compared commensal *E. coli* strains isolated from the livestock environment with *E. coli* strains responsible for clinical infections in livestock. We found that pathogenic isolates have significantly higher MIC values for GLY but not for RU, supporting the data described in the literature to some extent.

This is likely to be explained by superior stress responses in pathogenic bacteria (Chowdhury et al., 1996; McKellar and Knight, 1999). Therefore, the capacity to adapt to changes can lead to a decreased susceptibility (Chowdhury et al., 1996; Poole, 2012).

In contrast, some ingredients in the formulation seem to eliminate the advantages pathogenic bacteria have, with all isolates generally showing less sensitivity to RU.

In conclusion, higher MICs for GLY in pathogens can be a side effect of the overall benefits to adapt as mentioned before and does not necessarily imply resistance to glyphosate itself. A closer look into the genetics of the resistance mechanisms and the target structure of the herbicide are required for further studies.

### Host Impact

There are statistically significant differences between the host species of the *E. coli* strains, both for GLY and RU in all tests and calculated models. Bacteria isolated from poultry showed higher MICs compared to isolates from cattle and pig.

Farm animals have individually composed feed, and accordingly, different levels of glyphosate exposure. Herbicide residues in feed can lead to the exposure of livestock-related bacteria to glyphosate and other compounds of formulations.

Little data is available about the amount of residues in feed, however, imported soybean meal seems to be the main source (von Soosten et al., 2016). Glyphosate has been found in poultry and cattle feed in Germany (Shehata et al., 2014) and in a study in cattle feed (Schnabel et al., 2017).

Poultry are typically fed with corn, wheat and barley, often supplemented with soy as a protein source. However, soy is also commonly used in pig and cattle feed. Therefore, exposure to glyphosate was possible for all hosts, though concentrations in the environment are considerably lower than in the conducted experiment.

Even though statistical analysis revealed MIC differences in the active ingredient and the formulation, there is currently no explanation for this. Data about residues in the feed and possible glyphosate exposure are lacking. Overall, reasons for varying susceptibility of *E. coli* isolates for glyphosate between the host species have to be elucidated in further investigations.

### Extended Spectrum Beta Lactamase (ESBL)/Non-ESBL

In our dataset, non-ESBL isolates had higher MIC values for GLY, whereas for RU no difference between ESBL and non-ESBL could be observed.

Although ESBL isolates have a resistance to β-lactam antibiotics, it was not accompanied by a higher tolerance of glyphosate. On the contrary, the MIC for GLY is lower in ESBL isolates. This is likely to be explained by the very different mechanisms behind these two resistances. Glyphosate affects the shikimate pathway and disrupts the formation of aromatic amino acids necessary for bacterial protein synthesis (Steinrücken and Amrhein, 1980; Herrmann, 1995), whereas in ESBL 3rd and 4th generation β-lactam antibiotics are hydrolyzed (Pfeifer et al., 2010).

However, non-target site resistance can further affect other antimicrobials, as seen for example in biocide-antibiotic cross-resistances or cross-tolerance (Poole, 2012; Capita et al., 2014). Exposing *E. coli* to sub-lethal glyphosate concentrations in form of the formulation Roundup weed killer changed antibiotic susceptibility in both directions (Kurenbach et al., 2015) and adaptive resistance was mostly obtained through efflux pumps (Kurenbach et al., 2017). On the contrary, exposure to the biocide triclosan had no effects on unrelated antimicrobials (Ledder et al., 2006).

Different MICs could further be explained by fitness costs, which can accompany antibiotic resistances in bacteria (Melnyk et al., 2015).

Finally, a distortion in our isolate selection, as 12 of 15 pathogenic poultry isolates are non-ESBL (with higher MICs in pathogenic and poultry isolates as mentioned above) could explain the difference. However, the difference is not only present in the non-parametric test but indeed supported by the statistical model.

The surfactants present in RU could compensate for differences between ESBL and non-ESBL strains, explaining the similar higher MICs for the formulation.

In order to gain more clarity on the link of antibiotic and glyphosate tolerance, future studies should as well investigate effects of non-target resistances and include further antibiotics (e.g., tetracycline, macrolides, or aminoglycosides).

In conclusion, we conducted a large-scale screening for GLY and RU susceptibilities in 238 isolates of *E. coli*. We found small but statistically significant differences between the tested formulation RU and the pure glyphosate salt as well as between poultry and other host animals with higher MIC values in Roundup and poultry. Furthermore, for glyphosate, we observed differences between non-ESBL and ESBL, and between pathogenic and commensal isolates (higher MICs in the former group). The difference between recently sampled isolates and the historic ECOR collection from 1984 was only found when the Mann-Whitney-U test for GLY was applied, but this finding was not confirmed by the modeling.

While this pilot screening yielded intriguing results indicative of the relationship between different groups of *E. coli* and changes in sensitivity to GLY/RU, further detailed investigations are required. These would ideally include inter-laboratory repetitions on a larger number of isolates to determine the precision of the susceptibility testing, and sequencing of the isolates with the MIC values above the cut-off, as well as the analysis of their gene expression.

Importantly, further investigations are needed to determine whether the observed differences are due to glyphosate use and/or application of antibiotics, other biocides and heavy metals or inter-strain diversity (Moorman et al., 1992).

Furthermore, investigations into whether the presence of glyphosate residues in feed leads to the accumulation of pathogenic bacteria in livestock animals or in livestock farms are currently ongoing.

## Author Contributions

KB performed the experiments, collected, analyzed, and interpreted the data, drafted the manuscript and figures, with critical evaluation and support of all other authors. JP performed the experiments and collected the data. RM contributed to the statistical data analysis, and wrote sections of the manuscript. OM and UR conceived and designed the study, and critically revised the manuscript. All the authors approved the final version to be published.

## Conflict of Interest Statement

The authors declare that the research was conducted in the absence of any commercial or financial relationships that could be construed as a potential conflict of interest.
