# Supplementary material for: Minimum Inhibitory Concentration of Glyphosate and of a Glyphosate-Containing Herbicide Formulation for Escherichia coli Isolates – Differences Between Pathogenicand Non-pathogenic Isolates and Between Host Species
Source: Front Microbiol. 2019 May 3;10:932. doi: 10.3389/fmicb.2019.00932 (PMC6509229; doi:10.3389/fmicb.2019.00932)
Supplement: Supplementary file 1 [file Data_Sheet_1.docx]

Supplementary Material

**Supplementary Figure 1.** MIC for glyphosate isopropylamine salt in MH I (GLY MH I, black) and in MH II (GLY MH II, black with white stripes) and for the formulation Roundup LB Plus in MH I (RU MH I, white) and in MH II (RU MH II, white with black oblique stripes),respectively.

**Supplementary Table 1.** Effect of different parameters in MIC by means of univariable nonparametric Mann-Whitney-U test in MH II for glyphosate isopropylamine salt (GLY) and Roundup LB Plus (RU) as well as Wilcoxon test for differences between the MIC in MH I and MH II. Statistically significant *P*-values <0.05 are in bold. Parameters with higher MICs are underlined.

|  | | Mann-Whitney-U test | |
| --- | --- | --- | --- |
| comparison of | | GLY_MH II_ *P*-value | RU_MH II_  *P*-value |
| historic isolates | recent isolates | 0,051 | 0,961 |
| ECOR collection | Commensal *E. coli* | 0,062 | 0,871 |
| ECOR collection | Pathogenic *E. coli* | 0,114 | 0,791 |
| Pathogenic *E. coli* | Commensal *E. coli* | 0,994 | 0,614 |
| non-ESBL | ESBL | 0,237 | 0,215 |
| poultry | pig | **0,032** | **0,005** |
| poultry | cattle | **0,026** | **0,005** |
| pig | cattle | 0,965 | 0,939 |
|  |  | **Wilcoxon test** | |
| MH I | MH II | **<0,001** | **<0,001** |
|  |  |  |  |
| Median and Mode [mg/ml] | | 20 | 40 |
| MIC_95_ [mg/ml] | | 20 | 80 |
